# Feature Subset Selection for Malware Detection in Smart IoT Platforms

**DOI:** 10.3390/s21041374

**Published:** 2021-02-16

**Authors:** Jemal Abawajy, Abdulbasit Darem, Asma A. Alhashmi

**Affiliations:** 1 Cyber Security Research and Innovation Centre, Faculty of Science, Engineering and Built Environment, Deakin University, Geelong, VIC 3220, Australia; 2 Department of Computer Science, Northern Border University, 9280 Arar, Saudi Arabia; basit.darem@nbu.edu.sa (A.D.); asma.alhashmi@nbu.edu.sa (A.A.A.)

**Keywords:** Internet of Things, malware, Android OS, feature selection, machine learning, filter methods, malware detection, smartphones

## Abstract

Malicious software (“malware”) has become one of the serious cybersecurity issues in Android ecosystem. Given the fast evolution of Android malware releases, it is practically not feasible to manually detect malware apps in the Android ecosystem. As a result, machine learning has become a fledgling approach for malware detection. Since machine learning performance is largely influenced by the availability of high quality and relevant features, feature selection approaches play key role in machine learning based detection of malware. In this paper, we formulate the feature selection problem as a quadratic programming problem and analyse how commonly used filter-based feature selection methods work with emphases on Android malware detection. We compare and contrast several feature selection methods along several factors including the composition of relevant features selected. We empirically evaluate the predictive accuracy of the feature subset selection algorithms and compare their predictive accuracy and the execution time using several learning algorithms. The results of the experiments confirm that feature selection is necessary for improving accuracy of the learning models as well decreasing the run time. The results also show that the performance of the feature selection algorithms vary from one learning algorithm to another and no one feature selection approach performs better than the other approaches all the time.

## 1. Introduction

The Internet of Things (IoTs) has come to permeate all aspects of our life and IoT devices such as smartphones and smartwatches have become necessary in modern mobile-centric connected world. Android is an open-source operating system (OS) devised primarily for use in smart IoT devices. With 71.18% market share [1], Android is undoubtedly the leading operating system used in IoT devices such as smartphones worldwide. Permission-based models are used in the Android platform to protect the IoT devices from dangerous apps. However, this security model has proven to be inadequate for dealing with malware threats for IoT devices using Android OS [2]. Currently, malware targeting the Android platform far outnumbers all the other platforms and continue to rise considerably over the last few years [3]. For example, in 2018 Kaspersky detected about 5,321,142 malware samples from different families targeting Android platforms. Also, Android app distribution markets such as third-party markets and official Google Play Store market have become a haven for malware app distribution [4]. Specially, the third-party markets hosting up to 50% malware apps [5], tend to be replete with malicious apps. Although Google tries to weed out malware-infected apps from its market, the Google Play Store occasionally hosts malicious apps estimated to be up to 22% of the apps uploaded on the Google Play Store [6].

Android malware can infringe on users privacy, compromise data integrity and destroy sensitive information. With the fast evolution of Android malware, it is practically not feasible to manually detect malware apps in Android ecosystem. Similarly, the signature-based traditional classification methods are not effective [2] thus calling for an alternative approach that can quickly and effectively detect malware apps. As a result, machine learning algorithms are taking the centre stage to malware detection [2,7,8]. Machine learning based solutions rely heavily on extracting meaningful features from the Android apps for training the models [9]. Generally, static and dynamic analysis methods are utilized to extract typical malware descriptive behaviour (i.e., features) from the raw data. These feature extraction methods normally generate very large high-dimensional, redundant and noisy features [10,11]. Some of the raw features offer little or no information that is useful to distinguish malware apps from benign apps and may even impact the performance of the malware detection methods [10,12,13,14]. As a result, automatic feature subset selection has become a key aspect of machine learning [15].

Feature selection algorithms select a subset of features from the original feature set, which are considered useful for training the learning models to obtain good results [2,10]. A growing number of Android malware detection models have applied different feature subset selection algorithms and have achieved good detection rates [8,16]. However, research on the usefulness of the state-of-the-art feature subset selection methods in the context of Android malware detection models have not received the attention it deserves [10]. To this end, we investigate the utility of the commonly used feature subset selection approaches for malware detection in Android platforms. We analyse the feature selection methods with the goal of finding out: (i) the order in which they select the subset features, (ii) the usefulness of the selected features on the performance of the learning models, (iii) similarities between the various feature selection methods with respect to feature ranking, and (iv) the direct influence of varied feature length on the learning model classification accuracy. The contributions made in this paper can be summarized as follows:

- We formulate the feature selection problem as a quadratic programming problem; and analyse how different feature selection methods work and how they are used in Android malware detection models,
- We compare and contrast several commonly used filter-based feature selection methods along several factors,
- We analyse the requested permissions distribution of the samples and the composition of the relevant feature subsets selected by the feature subset selection algorithms thoroughly to discover the usefulness of the feature subsets,
- We empirically evaluate the predictive accuracy of the feature selection techniques using several learning algorithms that do not perform feature subset selection internally, and
- We demonstrate the usefulness of feature selection in Android malware classification systems.

We organise the remainder of the paper in the following manner: In Section 2, the problem overview is given. Section 3 will review some related work while Section 4 highlights the model used in this paper. Experimental evaluation is discussed in Section 5. The conclusion remarks and future work are discussed in Section 6.

## 2. Problem Overview

Quality features are crucial for building effective machine learning based classification models. This is because raw features extracted from Android apps for the purposes of training the models are very large. For example, Su et al. [11] extracted more than 50,000 different features from an Android app. Typically, some of these raw features are key in differentiating malware from benign apps while others are not [17]. Also, too many features lead to the model complexity and to the “curse of dimensionality” problem [17]. Moreover, these features tend to be high-dimensional [18] and replete with a large number of redundant features that may not be relevant to exclusively differentiate Android malware from benign apps [11]. It is not useful for machine learning algorithms to directly handle high-dimensional data [19]. This is because such data normally contains significant noises and irrelevant features that add little or no value to the performance of the learning algorithms. Therefore, these unwanted features should be removed from feature subsets to be used in training the learning models.

The feature selection problem can be formulated by using quadratic programming. Specifically, given a dataset of n training samples $\left\{\left(F_{i},\;l_{i}\right)\;|\;1\leq i\leq n\right\}$, where $l_{i}$ is the target classification label and $F_{i}=\left\{f_{ij}\;|\;1\leq j\leq m\right\}\;\in {\mathbb{R}}^{m}$ is a vector of m raw features such that n≪m. The feature subset selection problem is to select a subset of the original features from the observation space ${\mathbb{R}}^{m}$ for use in training the learning algorithms. This problem can be formally stated as a quadratic programming optimization problem as follows [20]:

(1) $$\underset{X}{\max}\left(X\;\left(\frac{X^{T}\times S}{2}-F^{T}\right)\right)\;$$

Such that

(2) $$x_{i}\geq 0\;\forall i\;1,\cdots ,m\;$$

(3) $$\overset{m}{\underset{i=0}{\sum }}x_{i}=1\;\left(3\right)$$

The above formulation considers the relevance of the features to the class label whereas redundancy between the features is penalized. The parameter $S\in {\mathbb{R}}^{m\times m}$ in the above equation is a similarity matrix used for capturing features redundancy; the parameter $F\in {\mathbb{R}}^{m}$ quantifies the level of correlation between each feature and the target class label (i.e., captures how relevant the feature is). The entries in $X=\left\{x_{1},\;x_{2},\cdots ,x_{m}\right\}$ represent the weight of each feature as computed by F. The constraints in Equations (2) and (3) enforce the weight of each feature (i.e., $x_{i}\in X$) should be non-negative must add up to one. Normally, features with weights above a given threshold are considered useful features and selected for subsequent training of the learning algorithms.

Although feature subset selection is important in machine learning, finding the best subset features from the original features is known to be an NP-hard optimization problem [20]. This is because the feature selection algorithm has to examine a total of $2^{m}-1$ candidate subset of features. As the number of m increases, it quickly become evident that examining all of the features exhaustively cannot be done in practice. An exhaustive search for the best possible features from the original feature sets is practically unfeasible in most cases. Solving the problem, even for a modestly large m, is computationally impracticable. As a result, many heuristic approaches have been proposed in the literature to solve this problem.

## 3. Related Work

Feature subset selection is among the top fundamental challenges in machine learning arena. The problem has continued to draw an increasing attention from researchers and practitioners alike [7,8,10,18,21,22,23,24,25,26,27,28]. Although choosing a subset of features from the original features is a combinatorial problem, many suboptimal heuristics have been put forward and used in various domains, which include the chi-squared based feature subset selection [7,8,10], the analysis of variance (ANOVA) [7,8,10], mutual information [7,23,29] and information gain [18,25,26,27]. Many studies have shown that feature selection approaches that select good feature subset will have significant impact on reducing the complexity in processing by eliminating unimportant features and enhance the performance of the learning models [24,30]. For example, Aonzo et al. [30] demonstrated that small number of features are enough for a very good classification. They considered the most significant features extensively used in prior research. They selected a small number of features from the list of most important features and show that the small subset features they are enough for a very good classification.

Exiting feature selection approaches are organized into filters, wrapper, embedded and hybrid methods [15,16]. The filter methods select a subset of features without altering their original representation. A statistical criterion is used in filter-based feature selection techniques to assess the relevance of the features. The selected features can be used by any learning methods because the selection is not tied to any machine learning method. In contrast, feature selection in the wrapper-based approach involves a classification model to assess the suitability of the features [15]. Several authors empirically compared representatives of filter, wrapper, and embedded feature selection methods using simulated data. Bolón-Canedo et al. [19] analyzed the seven filter-based feature selection methods, two wrapper feature selection methods, and two embedded feature selection methods using synthetically created microarray data sets under four machine learning classifiers. Similarly, Wah et al. [31] investigated how the filter methods compare with the wrapper methods in terms of the classifier accuracy. The authors compared two filtering methods, namely the correlation based and the information gain feature selection against two wrapper methods, namely the sequential forward and sequential backward elimination methods. The feature selection methods are tested using both artificial data sets and real data sets using logistic regression as a classifier. Xue et al. [32] compared the filter-based feature subset selection and wrapper-based feature subset selection methods with respect to classification accuracy and execution time. These works show that the wrapper methods generally achieve better classification performance than the filter method but much slower than the filter method.

Alazab [8] discussed a supervised machine-learning algorithm for Android malware detection using various feature sets that are generated using the Chi-Square and one-way ANOVA feature subset selection methods. The detection accuracy of ten supervised machine-learning algorithms were evaluated to identify the most reliable classifier for malware detection. The model with feature subsets produced by Chi-Square was found to have a higher detection accuracy than the feature subsets produced by ANOVA. Wang et al. [7] experimented with three filter-based feature selection methods, namely Mutual Information, Chi-Squared and one-way ANOVA to avoid overfitting of their model. The authors used the selected features to train linear regression (LR) models with different numbers of selected top features, and thus compare their performances and the performance with full feature sets. The main tenet of the above studies is to develop Android malware detection, which differs from the main objective of our work.

Masabo et al. [10] proposed a New Feature Engineering (NFE) feature subset selection method that utilizes the domain knowledge of the data to create feature subsets. The authors assessed the power of NFE on feature selection by comparing it against one-way ANOVA, Recursive Feature Elimination (RFE), and PCA using KNN and Linear Discriminant Analysis and Gradient Boosting Classifiers. Thus, the main focus of this work is to evaluate the performance of NFE as compared to ANOVA, RFE and PCA on feature selection. The authors used 30 most discriminating features to assess the feature subset selection models and shown that NFE outperforms the other approaches in terms of precision, recall, and F score. In contrast, we compare and contrast several feature selection methods along several factors including the composition of relevant features selected. Moreover, PCA transforms the original features and it is not really good to compare it with other approaches that do not transform the original features.

Wang et al. [33] discuss one-class classification methods for detecting zero-day Android malware attacks using Intra-Class Distance (ICD) feature selection method. The one-class classification methods use benign samples only to construct the detection model as opposed to the two-class models that use both benign and malware samples. In order to justify the use of ICD, the authors compared it against PCA and Pearson Correlation Coefficient methods using the Gauss Distribution and ν-SVM classifiers. It is shown that Pearson Correlation Coefficient has a significantly poorer classification and runtime performance as compared to the ICD and LS. Although ICD and LS have comparable classification performance, ICD has significantly lower runtime than the other models.

Mas’ud et al. [22] investigated the use of several different feature selection methods in optimizing the n-gram system call sequence feature in classifying benign and malicious mobile application. The n-gram system call sequence can generate a large number of features to be used in the classification and can contribute to the degradation of classification performance. Several filter and wrapper feature selection methods are selected, and their performance analyzed. Four different filter methods, namely Correlation-based Feature Selection (CFS), Chi Square (CHI), Information Gain (IG), ReliefF (RF) and one wrapper method with a Linear SVM classifier (WR) are chosen to be evaluated in this paper. The feature selection methods are evaluated based on the number of feature selected and the contribution it made to improve the True Positive Rate (TPR), False Positive Rate (FPR) and Accuracy of the Linear-SVM classifier in classifying benign and malicious mobile malware application.

Mahindru and Sangal [24] discuss a Least Squares Support Vector Machine (LSSVM)- based malware detection system. The authors analyzed various feature selection methods for the purpose of selecting the relevant features. These feature selection approaches include Pearson’s correlation coefficients, Chi-Squared, Rough set analysis (RSA), Information-gain, Consistency subset evaluation and PCA. Empirical result reveals that the model using the Rough Set Analysis (RSA) feature selection achieved better results when compared to the other feature subset selection methods. Bommert et al. [34] comprehensively compared various filter-based feature selection techniques available on different toolboxes. It is concluded that there is no single feature subset selection that is superior to others all the time but some of the methods always perform well on many of the data sets.

Wang et al. [29] used the mutual information, Pearson correlation coefficient, and T-test feature subset selection with the aim of understanding the possible risks posed by Android permissions. The permissions are ranked based on their risks. In order to determine risky permission subsets, two different methods, namely sequential forward selection as well as the PCA (principal component analysis) are deployed. The experimental results, using several machine learning (SVM, decision trees, and random forest) algorithms, the authors show that risky permissions as features offer satisfactory performance with a detection rate as 94.62% with a false positive rate as 0.6%. This work is focused on identifying risky permissions and its use in Android malware detection. In contrast, we are focused on feature subset selection algorithms use in Android malware detection.

Wang and Li [35] used PCA, Correlation, Chi-square and Information Gain feature selection methods for the sake of dimension reduction. The authors examined the Android kernel features to identify Android malware. A Weight-Based Detection (WBD) approach is proposed to differentiate between Android malware and benign apps using Decision Tree, Naive Bayes, and Artificial Neural Network machine learning algorithms. The authors used 112 multiple dimensional kernel features of tasks and processes where the four feature selection methods. Vinod et al. [36] examined system calls for Android malware detection. The authors compared five feature selection methods for reducing higher dimensional system call set. The five feature selection methods are the symmetric uncertainty, information gain and principal component analysis (PCA), Absolute Difference of Weighted System Calls (ADWSC) and Ranked System Calls using Large Population Test (RSLPT). The last two methods are proposed by the authors whereas the other three methods are used as a benchmark to demonstrate the effectiveness of ADWSC and RSLPT feature selection methods.

There are many survey papers on feature selection methods [37,38,39,40]. In Wang, et al. [37], a systematic survey of the feature subset selection techniques and the features used in exiting literatures for Android malware detection is discussed. A taxonomy of existing features and feature selection methods is presented, and the issues of creating features for malware detection are highlight. The authors concluded that there is a need for further work that explore and refine well-discriminated features from numerous features extracted from Android apps. Feizollah et al. [38] analyzed about 100 publications with respect to feature selection in Android malware detection. The authors reported that feature selection algorithms were not exhaustively investigated in prior articles.

The prior works are either focused on non-malware related fields [19,30,31] or compare approaches that transform the original features against approaches that does not [10] or use classification methods that perform embedded selection of the feature subsets such as random forests [41] and gradient boosting [39] or use them as a validation of a newly proposed feature subset selection methods [10]. As noted in [19], it is essential that the efficacy of the feature subset selection is examined and verified on different situations and platforms such as Android malware classification. Also, classification methods that perform embedded selection of the feature subsets will not be able to explicitly quantify the influence of the feature selection methods. There is limited research in comprehensive analysis of feature selection methods for Android platform that avoids the above shortcomings. In this paper, we avoid classification algorithms with in-built feature subset selection. Also, we focus on filter-based feature subset selection algorithms since this class of feature selection methods have lower computational complexity as compared to the wrapper-based approaches. Moreover, research on malware detection in Android platform mostly deploy filter-based methods to select the subset of feature for training the learning algorithms [7,8,18,26,42]. Further, filter-based methods assess the suitability of features solely on statistical criterion and thus can be used in conjunction with any learning model.

## 4. Android Malware Detection Framework

**Figure 1:**
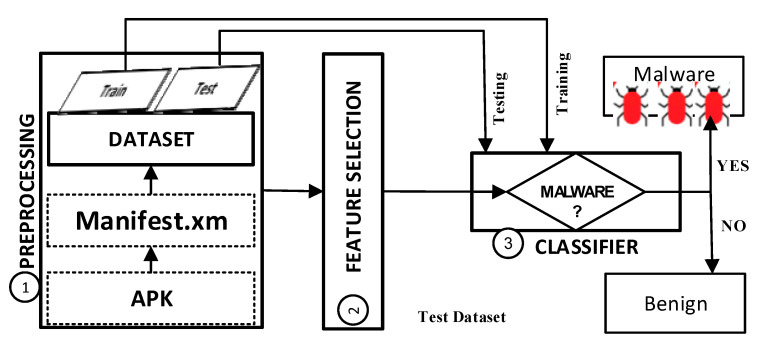
General framework for Android malware detection.

As shown in Figure 1, the general automated classification framework for Android malware detection consists of three general phases: (1) the sample dataset preprocessing (includes feature extraction) phase, (2) a phase for selecting feature subsets, and (3) a classification phase. The detailed descriptions of the first phase is given in this section. The other two phases will be discussed in separate subsections.

### 4.1. Unpacking Files

APK (Android Package) files are used to install apps on Android devices. It is a zip compression package and contains all the necessary contents of an Android apps. We followed the standard procedure for unpacking APK file apps as in [8]. We sourced APKs from various sources, which includes AndroZoo [4] and Drebin sites. To ensure that they are goodware, we used VirusTotal to scan the dataset and validate them that they are clean.

### 4.2. Feature Extraction

Android uses several permissions to guard users’ sensitive information from Android apps on the smartphone [30,43]. Each permission is associated with a specific operation such as accessing secure sections of the API, contact lists and camera. Therefore, the apps that are granted a particular permission are allowed to perform the operations linked to that permission. As malware writers usually achieve their goals by exploiting Android permission, differentiating malware apps from benign apps through behavioral analysis of Android permissions has been the focus of numerous research [8]. Therefore, we used the Android app permissions as features from the malware and benign sample apps. AndroidManifest.xml file contains the permissions each Android app requested from the system. However, AndroidManifest.xml is a binary file and thus we have to first convert it to a plain text file. Then, we extracted the permissions requests from each app from the manifest file.

### 4.3. Feature Vectors

We use an n by m dimensional binary vector to represent the apps features. Given a set of apps, $A=\left\{a_{1},\;a_{2},\cdots ,a_{n}\right\}$, and a set of $F=\left\{f_{1},\;f_{2},\cdots ,f_{m}\right\}$ permissions, we created a features vector as follows:

(4) $$v_{ik}=\{\begin{array}{c} 1\;if\;app\;i\;request\;permissoin\;k\;\\ 0\;otherwise \end{array}\;$$

An example feature vector is shown in Table 1. We use Boolean values recorded in feature vector to refer to presence (1) and absence (0) of a permission in the apps. That is to say, if an app $a_{i}\in A$ requested a feature $f_{i}\in F$, the vector entry for this app will be 1. However, if a particular permission is not requested by an app, the vector entry will be 0 since the feature is not present in the app.

Generally, the last column of the feature vector holds the value of the target class label. For a malware app, the entry will be 1 and for benign apps the entry will be 0. In order to check the apps for the label (malware or not), we used the VirusTotal that incorporates more than 80 virus detection engines. The vector in Table 1 is used for training and testing the classifiers.

## 5. Feature Subset Selection Methods

Both the number and quality of the features used to train models to classify Android apps with respectable accuracy as a benign and malware are paramount. To this end, feature selection is used to weed out irrelevant, redundant, constant, duplicated, and correlated features from the raw features before training the models. A variety of filter methods for selecting the best features in Android malware detection frameworks have been widely deployed. In this section, we present detailed descriptions of the filter feature selection algorithms.

The basic tenet of filter-based feature subsets selection algorithms is that features that have a high correlation with the target are considered useful to enhance the training of the learning algorithms and subsequently improve the classification performance. Generally, filter methods are classified as univariate and multivariate methods. Univariate filter methods assess and rank a single feature at a time independently (i.e., the no evaluation for correlation among the features); multivariate filter methods assess the entire feature space while considering the relationships between the features. Multivariate filter methods are able to handle duplicated, redundant, and correlated features. In contrast, univariate methods do not consider the relationship between features and thus unable to handle duplicated, redundant, and correlated features. Each filter method uses a distinct performance parameter to rank the feature.

**Figure 2:**
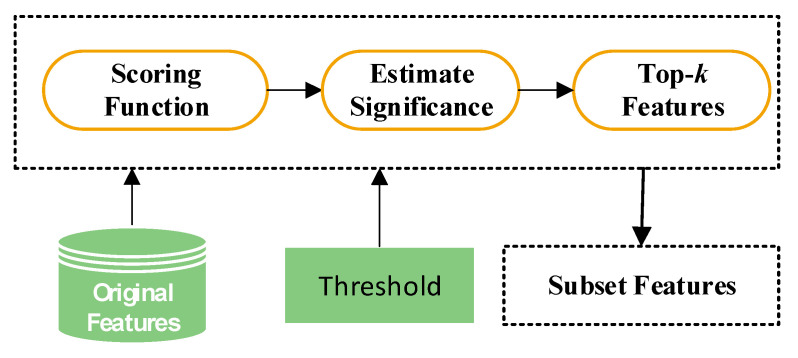
An architecture of filter methods for feature selection.

Generally, filter methods follow a typical scenario described in Figure 2. Given a set of n features,$\;F=\left\{f_{1},\;f_{2},\cdots ,f_{n}\right\}$, and the class label C∈{benign, malware} (as a target), the filter methods rank the F features based on certain criteria according to their degrees of relevance to the class label, and return the ranked features. Note that the features are judged solely based on the intrinsic characteristics of the features in relation to the target either individually or taking into account the statistical relationships between the features. A variety of scoring function is used to differentiate informative and discriminating features from less significant features. Normally filter methods perform statistical analysis such as correlation analysis or mutual information to assess and rank the features. The features with top rankings that is equal or exceed a threshold value are returned as the most suitable features while the rest are discarded.

Although there are many filter-based feature selection methods, we are considering only a subclass of filter-based that does not perform feature transformation. In other words, the selected features have not gone through any transformation and keep the semantics of the original features. Also, note that the features are selected independent of any learning algorithm. Table 2 compares the filter-based feature selection methods discussed in this paper in terms of univariate (UV) or multivariate (MV), the ranking used, the relationship between the feature and the target and the feature type supported. Para indicates if it is parametric (Y) or a non-parametric (N). The filter methods discussed here are capable of classifying data sets with either numeric features or categorical features. They can also be univariate (e.g., Pearson correlation coefficient) or multivariate (e.g., mutual information). The main difference between these two classes of filter methods is that the univariate (UV) filter methods do not consider interactions between the features whereas the multivariate (MV) methods do consider interactions between the features.

### 5.1. Pearson Correlation Coefficient

Pearson correlation coefficient is used in many Android malware detection methods [29,35], Pearson correlation coefficient uses statistical measures (i.e., linear correlation coefficient $\left(R_{xy}\right)$) to determine the strength of the correlation between a pair of variables X and Y. The linear correlation coefficient $\left(R_{xy}\right)$ can be computed as follows [29]:

(5) $$R_{xy}=\overset{n}{\underset{i=1}{\sum }}\frac{\left(x_{i}-\bar{x}\right)\times \left(y_{i}-\bar{y}\right)}{\sqrt{{\left(x_{i}-\bar{x}\right)}^{2}}\;\times \sqrt{{\left(y_{i}-\bar{y}\right)}^{2}}}$$

where n is the sample size, $x_{i}\in X$ and $y_{i}\in Y$ are the ith data values, and $\bar{x}$, $\bar{y}$ are the mean values. Equation (5) will result in $R_{xy}=\left\{-1,\;0,\;+1\right\}$, where the value of $R_{xy}$ is close to ±1 indicates that the correlation coefficient between a feature and the target class is high enough and the feature is selected. In contrast, if $R_{xy}$ is close to 0, the correlation coefficient is low, and the feature can be dropped.

### 5.2. Chi-Square

Chi-square is among the common feature subset selection algorithms used for malware classification in Android platform [8,36]. Chi-Square $\left(\chi^{2}\right)$ is used to determine whether the occurrence of a specific feature and the occurrence of a specific class label are independent from each other or not. Formally, $\chi^{2}$ for a given feature $f_{i}\in F$ is computed as follows [35]:

(6) $$\chi^{2}=\sum^{\;}\frac{{\left(f_{i}-C\right)}^{2}}{C}\;,\;1\leq i\leq n\;$$

Normally, the features are ranked in ascending order following the calculation of $\chi^{2}$ for each feature. The higher the $\chi^{2}$ score the more the feature $\left(f_{i}\right)$ and the class (C) are considered as dependent and the features that are highly dependent on the occurrence of the class label are considered as good candidates while those that are independent considered as a noninformative for classification purposes thus can be dropped.

### 5.3. Analysis of Variance (ANOVA)

The analysis of variance (Anova) is used in several studies as a feature selection method [7,8,10]. For feature, $f_{i}\in F$, the degree of dependency with the class label is estimated and the feature is given a score based on F-statistics. The f-score is computed as the ratio of within group variance and between group variance as follows:

(7) $$\mathrm{F-statistics}=\frac{variation\;between\;sample\;means}{variation\;within\;the\;samples\;means}\;$$

The variation between sample means (SSB) and variation within the means (SSW) samples are expressed as follows:

(8) $$\mathrm{SSB}=\underset{c_{k}}{\sum }\frac{N_{k}{\left({\bar{x}}_{k}-\bar{x}\right)}^{2}}{\left(m-1\right)}\;$$

(9) $$\mathrm{SSW}=\underset{c_{k}}{\sum }\underset{x_{i}}{\sum }\frac{N_{k}{\left({\bar{x}}_{ki}-{\bar{x}}_{k}\right)}^{2}}{\sum_{c_{k}}\left(N_{k}-m\right)}\;$$

where ${\bar{x}}_{k}$ is the sample mean, $\bar{x}$ is the class mean, m represent the total number of class labels, and $N_{k}$ is the number of class label $c_{k}$. The features are ranked in ascending order of their F-score and the top features that meet the selection criterion are identified for further use.

### 5.4. Information Gain

Given a feature F and a class label L (malware, benign), the information gain (*IG*) quantifies the amount of information feature F contributes to the accurate prediction of the class L. Formally, IG for a given feature F and a class label L is expressed as follows [35]:

(10) IG(L|F)=H(L)−H(L/F) 

where H(L) represents the prior uncertainty of L (i.e., entropy of a feature) and H(L/F) denotes the expected posterior uncertainty, which are computed as follows:

(11) $$H\left(L\right)=-\underset{i}{\sum }P\left(L_{i}\right)\cdot \log_{2}\left(F\left(L_{i}\right)\right)\;$$

(12) $$H(L|F)=-\underset{j}{\sum }P\left(F_{j}\right)\underset{i}{\sum }P\left(L_{i}|F_{j}\right)\cdot \log_{2}P\left(L_{i}|F_{j}\right)\;$$

where $P\left(L_{i}\right)$ and $P\left(L_{i}|F_{j}\right)$ refer to the prior probability of L, and the posterior probability of L given feature F respectively. Equations (11) and (12) give the entropy of *L* before and after observing *F*. The features are ranked in ascending order of their IG(F) and the top features that meet the selection criterion are identified for further use. Normally, the features with a high IG(F) value are taken to be relevant features, whereas those that do have a lower IG(F) value are considered not useful feature.

### 5.5. Mutual Information

Mutual information (MI) can measure the relevance of specific permissions by capturing a correlation between a given features F and a class label L based on how much information they share. Specifically, the mutual information between L and F can be measured by using the Kullback-Leiber divergence as follows:

(13) $$MI\left(F,L\right)=-\underset{x_{i}\in \left\{0,1\right\}}{\sum }\underset{c_{i}\in \left\{c_{0},c_{1}\right\}}{\sum }P\left(F=x_{i},L=c_{j}\right)\cdot \log_{2}M\frac{P\left(F=x_{i},L=c_{j}\right)}{P\left(F=x_{i}\right)\times P\left(L=c_{j}\right)}\;$$

where $P\left(L=c_{j}\right)$ is the occurrence rate of L with score $c_{j}\in \left\{0,\;1\right\}$, $P\left(F=x_{i}\right)$ is the rate of recurrence of F with score $x_{i}\in \left\{0,\;1\right\}$, and $P\left(F=x_{i},L=c_{j}\right)$ is the frequency count of F with value $x_{i}\in \left\{0,\;1\right\}$ in class $c_{j}$. The features are ranked in ascending order of their MI(F) and the top features that meet the selection criterion are identified for further use. Basically, the larger the value of MI(F), the greater the relationship between the *L* and *F*. But if MI(F)=0, then *L* and *F* are said to be independent (i.e., no correlation).

## 6. Performance Analysis

In this section, detailed descriptions of the learning models used, the data sets characteristics, the performance metrics used, and cross validation are discussed. Table 3 shows the naming convention used in the paper.

### 6.1. Experimental Setup and the Dataset

The experiment was performed on a 4th Gen. Intel Core i5 CPU with 12 GB of RAM running the Ubuntu 18.10 operating system. As in [43], we used WEKA 3.8, an open source platform, that supports many kinds of classifiers and different feature subset selection methods [44]. We used publicly available real-world dataset that includes 6190 benign apps from the Google Play Store and 5500 malware apps randomly sourced from VirusShare, Drebin and AndroZoo. The selected dataset provides an acceptable ground truth. This is because the data sources are relatively reliable, and many published works have used them. We also rigorously analysed the dataset using VirusTotal to ensure that they are exactly as we want them to be (i.e., malware or benign).

### 6.2. Performance Metrics

We use two metrics, namely accuracy and $F_{1}$ score for the purpose of assessing the efficacy of the models. These metrics are used in many studies including [10,24,29]. In this regard, accuracy can be formally expressed as follows:

(14) $$accuracy=\frac{\;correctly\;classified\;instances}{total\;number\;of\;instances}\;$$

The computed value will be in the range of [0, 1]. The closer the accuracy to 1.0, the higher the performance of the model.

$F_{1}$ is used to measure the precision and recall at the same time based on harmonic mean. It is defined as follows:

(15) $$F_{1}=2\times \left(\frac{preceision\times recall}{preceision+recall}\right)\;$$

where the ‘precision’ and ‘recall’ parameters are expressed as follows:

(16) $$preceision=\frac{True\;Positive\;\left(TP\right)}{True\;Positive\;\left(TP\right)+False\;Positive\;\left(FP\right)}\;$$

(17) $$recall=\frac{TP}{TP+False\;Negative\;\left(FN\right)}$$

### 6.3. Malware Classification Models

The learning models to assess the performance of the feature subset selection methods described in Section 4 includes the following:

- K-Nearest Neighbours (KNN) is a supervised machine learning algorithm commonly deployed in malware classification problem. KNN uses a majority vote to classify a new instance based on its K closest instances. We experimented with K equal to three.
- Support Vector Machines (SVM) is also a supervised learning algorithm and commonly used in malware classification problem. SVM uses a hyperplane to represent different classes and map a new instance to one of the hyperplanes.
- Naïve Bayes (NB) is another supervised learning classifier. NB is based on the Bayes theorem with conditional independence assumption. NB, based on the joint probabilities of sample observations and classes, attempts to approximate the conditional probabilities of the class given a new instance.
- Logistic Regression (LR) is a statistical model that uses a logistic function to assign observations to a discrete set of classes based on the probability theory.

The chosen classification methods are not only popular machine learning models but also, they do not perform embedded feature selection. These classification models are ideal for evaluating the direct impact of the feature selection algorithms as they are not capable of embedded feature selection.

### 6.4. Cross-Validation

Cross-validation is a practical approach commonly deployed to avoid the problem of overfitting when evaluating the effectiveness of classification systems [24,43]. To evaluate the models, the k-fold cross-validation method is used, where the dataset is divided into k equal segments. We used the k-1 sets to train the models and one set is used to test the models with. In the experiment, we set k = 20.

## 7. Results and Discussion

In this section, we analyze the feature subsets selected by the selection approaches in terms of the classification accuracy and F1-score. We also examine the execution time needed for feature subset selection.

### 7.1. Permission Analysis

**Figure 3:**
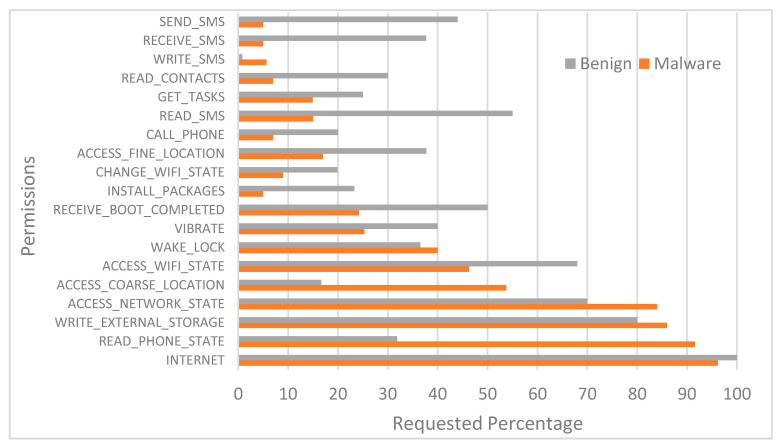
Distribution of most commonly requested permissions.

As noted earlier, each app requests a set of permissions necessary for it to operate. In this section, we analyse the distribution of the requested permissions in each app. For each permission requested by the apps, we obtained the percentage of the permissions usage in each class (i.e., benign and malware). Figure 3 shows the most frequently requested permissions by the samples in our dataset. The pattern of the permission requested by the apps is basically similar to the recent studies [29,45]. Although we can observe from the figure that few of the permissions (e.g., INTERNET and READ_PHONE_STATE) are the most widely requested permissions in the dataset by both benign and malware samples, prior research confirms that benign and malware apps requesting a similar set of permissions is very common.

Further, we can observe from the figure that benign apps and malware apps have dissimilar inclinations in regard to requesting different permissions. For example, we can see from the graph that malware apps ask for much more permissions than the benign apps (e.g., READ_PHONE_STATE). We can also observe from the graph that malware tends to request permissions that pose privacy or fraud risks (e.g., READ_PHONE_STATE and WRITE_SMS) as well as permissions from dangerous class than benign apps (e.g., ACCESS_COARSE_LOCATION and WRITE_EXTERNAL_STORAGE). Prior research has reported that more than 53% of malware apps tend to request dangerous permission such as READ_SMS whereas merely READ_SMS is requested by 1.36% of benign apps [29].

### 7.2. Feature Ranking Analysis

**Figure 4:**
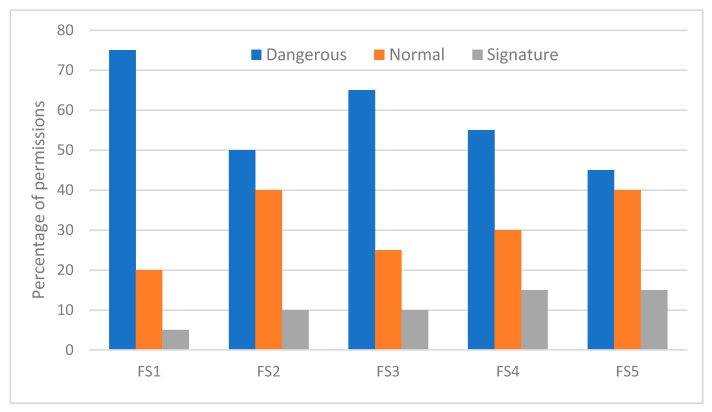
Distribution of the most frequently requested permissions with respect to their protection levels.

In order to determine the similarity between the feature subset selection approaches from the feature ranking point of view, we analyzed the methods by considering the top feature subsets recommended by the methods. Figure 4 shows the distributions of the top twenty features with respect to the permission protection levels (i.e., normal, dangerous or signature) for all feature selection methods. The top features selected from the dangerous permission level by the selection methods are in the range of 75% to 45% while between 15% to 5% of the top features are from the signature/system protection level class. FS1 selects 75% of the top features from the dangerous protection level class whereas FS5 selects 45% of the top features from the dangerous protection level class. Permissions selected by the FS3 method consists of 60% dangerous, 30% normal, and 10% signature levels.

Almost all feature selection methods rank SMS related permissions (e.g., READ_SMS, SEND_SMS, WRITE_SMS and RECEIVE_SMS) at the top. INSTALL_PACKAGES permission is also a dangerous permission with the tendency to be often requested by malware apps. Prior research shows that these SMS-related permissions are found frequently in malicious apps (Wang et al., 2017). The difference in the frequency count between the app classes in regard to READ_SMS, RECEIVE_SMS, and SEND_SMS is above 50% while it is in excess of 15% for WRITE_SMS permission. This clearly shows the difference between the malware and benign apps usage pattern of SMS related permissions. We also observe that some of the sensitive permission such as INTERNET are not among the top features suggested by the feature subset selection methods. This is because these permissions tend to be very commonly requested by both benign apps and malware apps.

**Figure 5:**
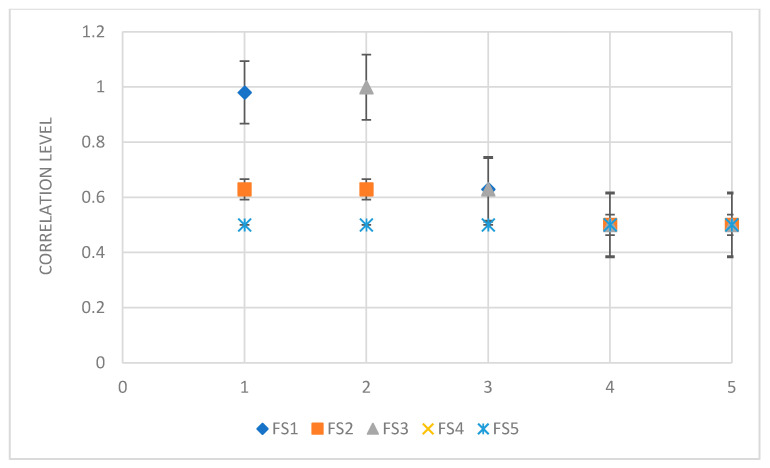
Similarity analysis between the feature selection methods with respect to the ranking.

In order to assess the degree of similarity between the feature subset selection methods with respect to the final ranking of the features, we performed a rank correlation coefficient measures using the Spearman correlation coefficient between the rankings. Figure 5 illustrates the similarity between the feature selection methods. An interesting observation is that the FS1 and FS3 seem to be quite closely resemble in terms of the ranking while the similarities between FS2 and FS3 are moderate. We find that 88.4% of features selected by FS1 and FS3 are the same, which make them very close.

### 7.3. Analysis of Feature Subset Selection Methods

In this section, we analyze the usefulness of the selected features in terms of their predictive performance on the classifiers. We use, as an important baseline, results with raw features (unfiltered) and compare it with the results of selected features.

#### 7.3.1. Impact of Feature Selection

**Figure 6:**
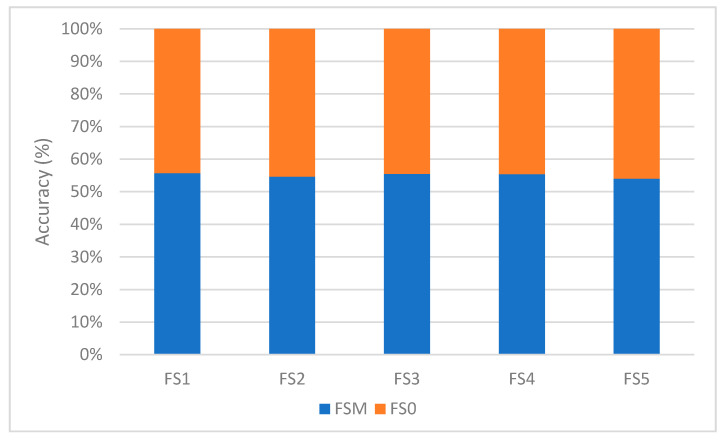
Performance with and without feature selections.

We now analyze the influence of feature subset selection methods on the Naıve Bayes classifier in terms of performance. As shown in Figure 6, a subset of feature (FSM) on average leads to a better performance than the case where feature selection is not deployed (FS0). This concurs with prior studies such as [8] in which Bayesian method combined with FS1 feature selection method yielded a better result as compared to Bayesian method without feature selection. This result can be explained by the fact that the unfiltered features contain various anomalies such as noise that impact the performance of a machine learning algorithm.

The feature selection methods select a mix of permission classes (i.e., normal, dangerous, signature). The result suggest that the classification accuracy could be greatly enhanced by blending features from all permission classes (i.e., normal, dangerous and signature).

#### 7.3.2. Classification Accuracy

**Figure 7:**
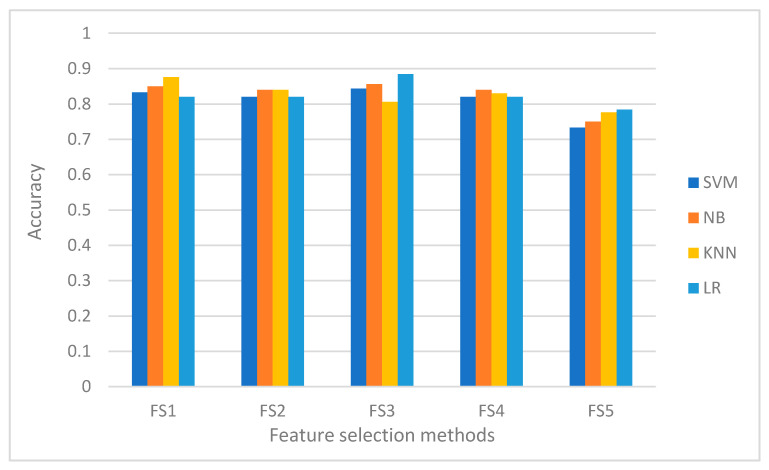
Performance of the five classifiers under the feature set produced by the five feature selection methods.

We now consider the predictive performance of the feature subsets recommended by the feature subset selection methods discussed in Section 4. We used the feature subsets with five machine learning models. Figure 7 shows the performance of the feature subsets under five different classifiers. From the results of the experiment, it is not possible to pinpoint a single feature subset selection method as winner. This is because none of the methods is always the best since there are many factors that influence the performance of the methods, which include the data sets used, the classifiers and the presence or exclusion of one or more relevant features. As can be observed from the result, feature subsets produced by a single method can lead to learning models with quite different predictive accuracy. For example, KNN performs better than SVM with FS1 while SVM performs better than KNN with FS3.

**Figure 8:**
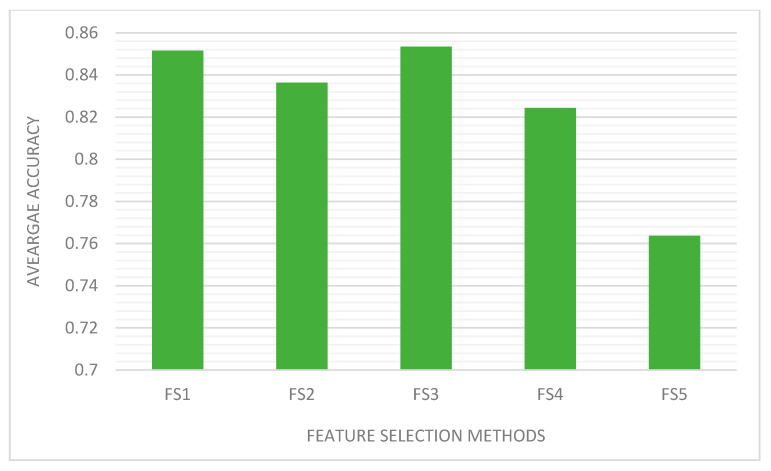
Performance of the five classifiers under the feature set produced by the feature subsets selection methods.

**Figure 9:**
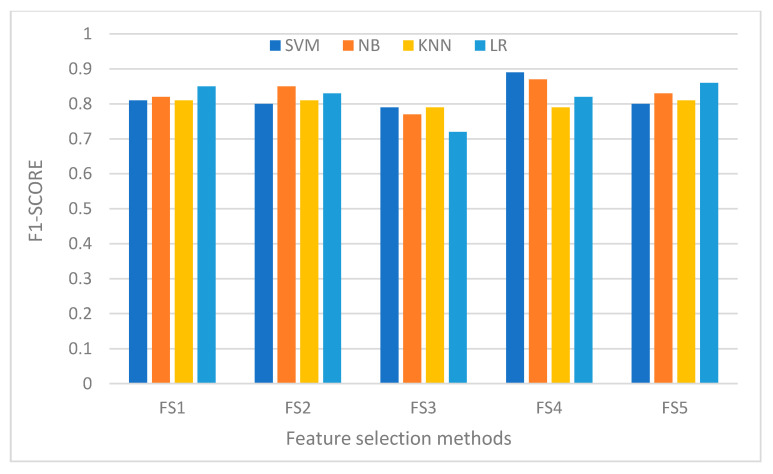
F1-score of the five classifiers under the feature subsets produced by the feature subset selections.

Overall, the result shows that FS1 and FS3 give better results as compared to the other feature subset selection approaches. Figure 8 shows that the mean accuracy of FS1 and FS3 are almost identical. This may be explained by the fact that these two feature selection methods share a significant number of features. They also have a good mix of risky permissions that are highly concentrated in malware apps, which enable the classifiers to discriminate well malware apps from benign apps by virtue of their appearance frequency. The result seems to support some of the published results [8]. Figure 9 shows that F-score of the five classifiers under the feature subsets produced by the feature subsets selection algorithms, which is consistent with accuracy figures.

### 7.4. Run Time Analysis

**Figure 10:**
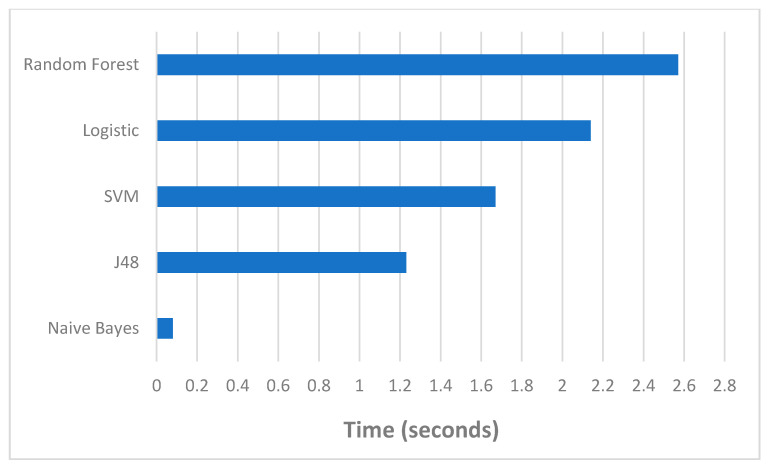
The run time for all data sets.

Figure 10 shows the execution time of the various models. We can observe from the figure that Naive Bayes (NB) is the fastest classifier. The KNN is naturally robust for small k value but it consumes large memory and computation time for training. The same issue with SVM regarding the need for large memory and thus more computation time for training. By filtering out extraneous as well as redundant features from the final features, we can reduce the running time of the learning algorithms significantly, the space complexity and yields a more general classifier [21]. Therefore, selecting the optimal set of features is necessary to reduce the space and time complexity as well as increase the accuracy or purity of classification [21,43].

## 8. Conclusions and Future Work

The detection of Android malware is a complex process that requires selecting a subset of discriminative features from the original samples. This study examined the feature selection methods commonly used in detection of malware in an Android platform with the goal of finding out the order in which they select the subset features, the predictive performance of the selected feature subsets on the classifiers, the similarities between the methods in terms of feature ranking and the influence of diverse feature length on classification accuracy. The study revealed that few of the permissions are popular among both malware and benign apps. The result of the study also shows that, on average all feature selection methods performed better than using the extracted features from the samples without filtering. Also, the chi-squared and the information gain approaches tend to do well as compared to others. However, these methods achieve high accuracy sometimes and perform poorly on other classifiers in the analysis. While filter-based feature subset selection techniques are effective computationally, they fail to handle issues such as multicollinearity that affects the accuracy of the filter methods. The problem with this is that a feature determined to be irrelevant on its own by a feature selection method maybe a significant predictor when it is combined with other features. Filter methods may miss such features since they normally do not address multicollinearity automatically.

We plan to extend this work in several directions. We plan to examine the scalability of the feature selection methods using very large datasets. Also, as Android malware apps are becoming increasingly sophisticated, our future work will focus on the need to characterize malware behaviours from different aspects. First, the current study shows that few of the permissions are popular among both malware and benign apps. Therefore, our future work will look at the implication of filtering out these common permissions on the performance of the detection accuracy of the classifiers. Also, the present study is solely based on a single component of Android system (i.e., permission) feature. As a single feature type may not be able to detect malware with sufficient accuracy, our future work will examine some combinations of the permissions on the accuracy level of the malware detection models. In addition, the correlation between the requested permissions and the actually utilized permissions with respect to more precisely reveal the behavioral pattern of the malware apps will be our other future work. Including other features such as API will also be studied in the future. Another direction of feature work is to examine how the characteristics of data and the machine learning models used favor one feature selection over the others will be studied. The theoretical aspect of feature selection is also another work planned to be tackled in the future.

## Figures and Tables

**Table 1:** Feature vectors for $a_{1},\;a_{2},\cdots ,a_{n}$ apps.

Apps	$f_{1}$	$f_{2}$	$f_{3}$	$f_{4}$	⋯	$f_{m}$
$a_{1}$	1	0	0	1	⋯	1
$a_{2}$	1	1	1	0	⋯	1
⋯	1	0	0	1	⋯	1
$a_{n}$	1	0	1	0	⋯	1

**Table 2:** Comparison of filter-based feature selection methods.

Filter	Variate		Para		Ranking	Relation		Feature Types
	UV	MV	Y	N		L	NL	
Pearson	√	×	√	×	$R_{FL}$	√	×	category
Information Gain	×	√	×	√	IG(L|F)	√	√	category
Anova	√	×	×	√	F-score	√	×	numeric or binary
Chi-Square	√	×	√		$\chi^{2}$	√	×	category
Mutual Information	×	√	×	√	MI(F,L)	√	√	category

**Table 3:** Naming conventions used in the paper.

Symbol	Corresponding Name	Symbol	Corresponding Name
FS1	Chi squared	FS4	Pearson Correlation Coefficient
FS2	Mutual information	FS5	Analysis of variance (ANOVA)
FS3	Information gain	FS0	All extracted features
KNN	K Nearest Neighbours	SVM	Support Vector Machines
NB	Naïve Bayes	LR	Logistic Regression

## Data Availability

We used publicly available real-world dataset from the Google Play Store, VirusShare, Drebin and AndroZoo.
